# Optical Coherence Tomography Predictors of Anatomical and Functional Recovery After Macular Hole Surgery

**DOI:** 10.1155/joph/5093898

**Published:** 2026-10-08

**Authors:** Ahmet Mehmet Somuncu, Mehmet Kola, Hidayet Erdöl, Büşra Köse, Mahmut Cenap Esenülkü

**Affiliations:** ^1^ Department of Ophthalmology Trabzon University Kanuni Training and Research Hospital Trabzon Türkiye; ^2^ Department of Ophthalmology Faculty of Medicine Karadeniz Technical University Trabzon Türkiye ktu.edu.tr

**Keywords:** macular hole, optical coherence tomography, retinal layers, visual acuity, vitrectomy

## Abstract

**Purpose:**

To examine whether preoperative optical coherence tomography (OCT)–derived macular hole parameters and postoperative outer retinal restoration are associated with anatomical closure and visual recovery after macular hole surgery and to determine whether preoperative geometry and preoperative symptom duration predict different outcome domains.

**Methods:**

This retrospective study included 48 eyes of 45 patients who underwent surgery for macular hole between January 1, 2013, and August 1, 2019, and completed at least 6 months of follow‐up. Surgery consisted of 23‐gauge pars plana vitrectomy (PPV) with internal limiting membrane (ILM) peeling; an inverted ILM flap was additionally used in 18 eyes (37.5%) and autologous blood in 4 eyes (8.3%). Combined phacoemulsification was performed in 12 eyes (25.0%). Best‐corrected visual acuity (BCVA) was recorded preoperatively and at Postoperative Months 1, 3, and 6. Preoperative OCT measurements included minimum hole diameter, basal hole diameter, hole height, macular hole index (MHI), tractional hole index (THI), diameter hole index (DHI), and hole form factor (HFF). Between‐group differences are reported with Cohen’s *d* and 95% confidence intervals (CIs), and a multivariable linear regression model was constructed for Month‐6 BCVA.

**Results:**

The cohort comprised 23 women (51.1%) and 22 men (48.9%) with a mean age of 68.4 ± 8.7 years. Anatomical success was achieved in 38 eyes (79.2%), and functional success in 36 eyes (75.0%). BCVA improved significantly through postoperative Month 6. Larger minimum and basal hole diameters were associated with worse postoperative BCVA, whereas higher MHI and THI values were associated with better postoperative BCVA. Eyes with preserved external limiting membrane (ELM) and/or ellipsoid zone (EZ) integrity at postoperative Months 3 and 6 had significantly better visual outcomes. Symptom duration was the dominant correlate of anatomical closure, and its association with BCVA strengthened across follow‐up (*r* = 0.523 preoperatively to *r* = 0.808 at Month 6) while that of THI weakened over the same interval. HFF was the only preoperative geometric parameter that differed between closure patterns (*d* = 0.86; 95% CI for the mean difference, 0.024 to 0.272; *p* = 0.036), yet it showed no association with BCVA at any time point. In a multivariable analysis, preoperative BCVA and Month‐6 EZ integrity were the only independent correlates of Month‐6 BCVA (adjusted *R*‐squared = 0.76).

**Conclusions:**

Preoperative OCT morphology is clinically informative in macular hole surgery. Minimum and basal hole diameters, MHI, and THI may help estimate postoperative visual recovery, while restoration of the ELM and EZ appears closely related to better functional outcome. In this cohort, the determinants of the two outcome domains were largely dissociable: preoperative geometry chiefly tracked functional recovery, whereas symptom duration dominated anatomical closure. HFF behaved as a marker of outer retinal restoration rather than of visual acuity.

## 1. Introduction

Macular hole is a vitreoretinal interface disorder in which the neurosensory retina is lost across its full thickness at the fovea. Patients notice blurred central vision, a central scotoma, and distortion. Most holes are idiopathic. They arise more often in women and are usually diagnosed in the sixth or seventh decade of life, and although the disease is typically unilateral, a substantial minority of patients go on to develop involvement of the fellow eye [1–4].

Anatomical success has improved considerably since pars plana vitrectomy (PPV) was introduced for idiopathic macular hole. Most centers now combine vitrectomy with internal limiting membrane (ILM) peeling or a flap technique and gas tamponade, and postoperative positioning remains part of the routine in many of them [5–7]. Visual recovery after a hole has closed is another matter. It still varies widely from eye to eye, which is why preoperative markers of the functional result retain their practical value.

Optical coherence tomography (OCT) resolves the retinal microstructure in cross‐section and allows the hole itself to be measured. Minimum diameter, basal diameter, and hole height can be read from a single scan, and several derivative indices follow from them: the macular hole index (MHI), the tractional hole index (THI), the diameter hole index (DHI), and the hole form factor (HFF). All have been investigated as predictors of surgical success [8–14]. A second line of work has followed the outer retina after surgery, where recovery of the external limiting membrane (ELM) and the ellipsoid zone (EZ) tracks functional recovery closely [15–21].

The prognostic value of minimum diameter, MHI, THI, and ELM/EZ restoration is well established individually, and we do not present these associations as novel. Less is known about how the same markers behave relative to one another, and relative to nonmorphological variables, within a single consecutively managed cohort followed at fixed intervals. Three questions motivated the present analysis. Do preoperative geometry and preoperative symptom duration predict the same outcome, or do they separate along anatomical and functional lines? Do their associations hold steady after surgery, or does their relative weight shift as the outer retina reconstitutes? And does HFF, the earliest OCT index proposed for macular hole prognosis and the one most often reported as a weak correlate of visual acuity [13, 20, 22], relate instead to the structural quality of closure?

We therefore examined preoperative OCT morphology, symptom duration, and postoperative outer retinal restoration together, with anatomical and functional endpoints assessed in parallel at Months 1, 3, and 6. The aim was to describe how prognostic labor is divided among these variables, not to re‐establish any of them individually.

## 2. Materials and Methods

### 2.1. Study Design and Ethics

This retrospective observational study was approved by the Ethics Committee of the Faculty of Medicine, Karadeniz Technical University (Decision Date: November 22, 2019; Approval No. 24237859‐340). The possible risks and benefits of surgery were explained to all participants, and written informed consent was obtained in accordance with the Declaration of Helsinki.

### 2.2. Patients and Clinical Assessment

We reviewed the records of patients who underwent surgery for macular hole between January 1, 2013, and August 1, 2019, and who had at least 6 months of postoperative follow‐up. Forty‐eight eyes of 45 patients met these criteria. Each patient was examined preoperatively and again at postoperative Months 1, 3, and 6. Snellen best‐corrected visual acuity (BCVA) was converted to logarithm of the minimum angle of resolution (logMAR) units for statistical analysis.

The following preoperative variables were recorded: age, sex, laterality, etiology, symptom duration, visual acuity, lens status, and hole stage. Stage was assigned by the Gass classification, using fundus examination together with the OCT findings. Lens status was not an inclusion criterion. At presentation, 28 eyes (58.3%) were phakic without visually significant lens opacity, 9 eyes (18.8%) were pseudophakic, and 11 eyes (22.9%) had a coexisting cataract. We excluded eyes whose media opacity prevented adequate OCT acquisition, and eyes with any additional maculopathy (diabetic maculopathy, age‐related macular degeneration, or previous macular laser).

### 2.3. OCT Measurements and Observer Reliability

Preoperative OCT analysis included basal hole diameter at the level of the retinal pigment epithelium, minimum hole diameter defined as the shortest distance between the hole edges, hole height from the retinal pigment epithelium to the ILM plane, THI (hole height/minimum diameter), MHI (hole height/basal diameter), DHI (minimum diameter/basal diameter), HFF (sum of the hole arm lengths divided by basal diameter), operculum presence, cystoid changes at the hole edge, and epiretinal membrane presence (Figure 1). All scans came from the same spectral‐domain device (Optovue RTVue, RT100 software, Version 6.3; Optovue Inc., Fremont, CA, USA), using five horizontal and five vertical raster sections. One section was chosen as the most representative profile of the hole, and every measurement was made by hand with the device caliper software.

**FIGURE 1:**
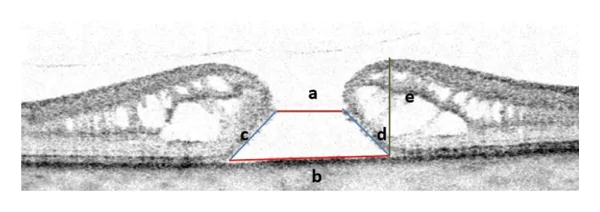
Schematic illustration of macular hole edge measurements and derived OCT indices. Minimum hole diameter (*a*), basal hole diameter (*b*), hole height (*e*), and hole arm lengths (*c*, *d*). MHI = *e*/*b*; THI = *e*/*a*; DHI = *a*/*b*; HFF = (*c* + *d*)/*b*.

A single observer (Ahmet Mehmet Somuncu) made all measurements without knowledge of the postoperative outcome. The study was retrospective and a remeasurement exercise could not be arranged, so no intra‐ or interobserver repeatability data are available; this is acknowledged in the Limitations.

### 2.4. Outcome Definitions

Postoperative anatomical success was defined as closure of the neurosensory retinal defect with disappearance of the perihole subretinal fluid. Functional success was defined as an improvement of at least two Snellen lines.

Closed holes were further classified by closure pattern at postoperative Month 6 according to the scheme of Kang et al. [23]: Type 1 closure denotes a closed hole without a residual foveal neurosensory defect, whereas Type 2 closure denotes a flattened but incompletely apposed hole in which a neurosensory defect persists. In the present cohort, this distinction was operationalized on OCT as the simultaneous restoration of ELM and EZ continuity across the fovea (Type 1) versus persistence of a discontinuity in either band (Type 2) (Figures 2 and 3). The criterion is thus the presence or absence of a residual neurosensory defect. It is distinct from the morphological U‐, V‐, and W‐shaped descriptors introduced by Imai et al. [24], which characterize foveal contour rather than defect status. One consequence needs stating. The operational criterion for closure pattern and the ELM/EZ integrity variable share the same anatomical substrate, so the two are not statistically independent. Postoperative BCVA is accordingly reported by the ELM/EZ status only and not additionally by the closure pattern, and the closure pattern analysis is confined to the comparison of preoperative parameters between the two patterns, which this dependency does not affect.

**FIGURE 2:**
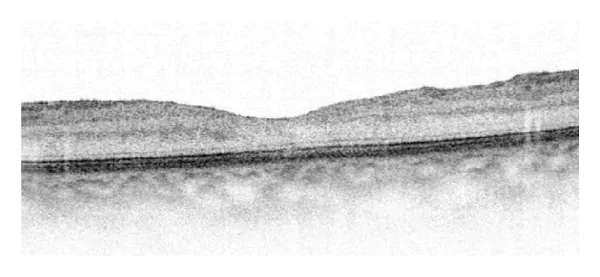
Representative OCT image of Type 1 closure: The hole is closed with restored continuity of the external limiting membrane and ellipsoid zone and no residual foveal neurosensory defect (arrowhead).

**FIGURE 3:**
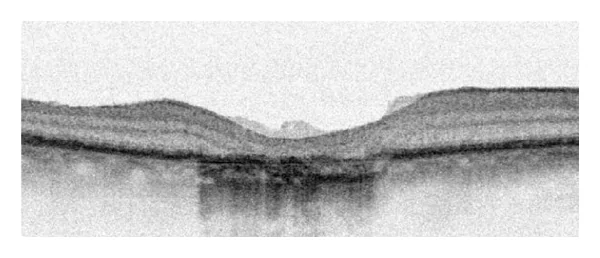
Representative OCT image of Type 2 closure: The hole edges are apposed, but a residual foveal neurosensory defect with outer retinal band discontinuity persists (arrowhead).

### 2.5. Surgical Procedure

All procedures were performed under general anesthesia by two surgeons (Mehmet Kola, Hidayet Erdöl) using a 23‐gauge three‐port transconjunctival system. After core vitrectomy, the posterior hyaloid was detached by active aspiration with the vitrectomy probe in eyes with an attached hyaloid. Diluted triamcinolone acetonide was injected over the macula to stain the ILM and any epiretinal membrane, and the ILM was peeled with 23‐gauge end‐gripping forceps out to the vascular arcades. Technique was not uniform across the study period. An inverted ILM flap was created and laid over the hole in 18 eyes (37.5%); the remaining eyes underwent conventional complete ILM peeling. Autologous whole blood, drawn intraoperatively under sterile conditions, was applied over the hole in 4 eyes (8.3%), following reports of blood‐derived adjuncts in macular hole surgery [25]. Tamponade was 15% perfluoropropane (C3F8) in 41 eyes (85.4%), filtered air in 4 eyes (8.3%), 22% sulfur hexafluoride (SF6) in 2 eyes (4.2%), and silicone oil in 1 eye (2.1%). Combined phacoemulsification with intraocular lens implantation was performed at the same session as the primary vitrectomy in 11 eyes (22.9%), and 1 further eye underwent combined phacoemulsification at reoperation, so that 12 eyes (25.0%) in total received combined lens surgery during the study period; no eye underwent clear lens extraction in the absence of lens opacity. Prone positioning was recommended for up to 1 week after surgery.

### 2.6. Statistical Analysis

Statistical analyses were performed with IBM SPSS Statistics Version 23.0 (IBM Corp., Armonk, NY, USA). Continuous variables are presented as mean ± standard deviation (SD), minimum, and maximum, while categorical variables are presented as counts and percentages.

Normality was assessed with the Shapiro–Wilk test. Two independent groups were compared with Student’s *t*‐test where parametric assumptions held and with the Mann–Whitney *U* test where they did not. Repeated BCVA measurements were not normally distributed, so within‐group comparisons used the Wilcoxon test. Comparisons across more than two independent groups used the Kruskal–Wallis test, followed by Bonferroni‐adjusted post hoc testing when appropriate. Ordered trends across Gass stages were additionally assessed with the Jonckheere–Terpstra test. Categorical variables were analyzed with the chi‐square or Fisher exact test (Fisher–Freeman–Halton exact test for tables larger than 2 × 2 with small expected counts), and associations between quantitative variables were assessed with Spearman correlation analysis. A two‐sided *p* value below 0.05 was considered statistically significant.

The cohort is modest, so *p* values are not interpreted in isolation. Every two‐group comparison of preoperative OCT parameters is reported with a standardized effect size (Cohen’s *d*) and a 95% confidence interval (CI) for the mean difference alongside the *p* value [26]. A nonsignificant result whose CI spans both zero and a clinically relevant difference is treated as inconclusive rather than as evidence of no association. The three qualitative preoperative features (operculum, epiretinal membrane, and cystoid changes at the hole edge) were compared across closure patterns and outcome groups with the Fisher exact test, with odds ratios and 95% CIs estimated using the Haldane–Anscombe correction where a zero cell was present.

A multivariable linear regression model was fitted with Month‐6 BCVA (logMAR) as the dependent variable. To respect the recommended limit of approximately 10 observations per predictor [27], the model was restricted to four covariates chosen a priori: preoperative BCVA, symptom duration, minimum hole diameter, and Month‐6 EZ integrity. Multicollinearity was assessed with variance inflation factors (VIFs). MHI and THI are algebraic functions of hole height and hole diameter, so they were not entered alongside the diameters but were tested in a separate secondary model. No logistic model was attempted for anatomical closure: only 10 eyes failed to close, and the resulting events‐per‐variable ratio would not support a stable fit. The discriminative capacity of symptom duration for anatomical closure was instead evaluated by receiver operating characteristic (ROC) analysis, with the area under the curve (AUC), its 95% CI computed by the DeLong method, and the Youden‐optimal cutoff.

Three patients contributed both eyes (3 of 45 patients, 6 of 48 eyes). Eyes of the same patient are not independent, so the primary analyses were repeated in a sensitivity analysis restricted to the first‐operated eye of each bilateral patient (45 eyes); the direction and significance of the principal findings were then compared with those of the full cohort.

## 3. Results

### 3.1. Baseline and Operative Characteristics

Forty‐five patients (48 eyes) were studied, 23 of them women (51.1%) and 22 men (48.9%). Mean age was 68.4 ± 8.7 years (range, 28–81 years) and mean symptom duration 6.8 ± 5.3 months (range, 2–24 months). Most holes were idiopathic (44 eyes, 91.7%); 2 eyes (4.2%) were traumatic and 2 eyes (4.2%) followed retinal detachment. By Gass staging, there were 3 Stage 2 eyes (6.3%), 22 Stage 3 eyes (45.8%), and 23 Stage 4 eyes (47.9%). Baseline characteristics are summarized in Table 1; comorbid systemic conditions, which were not related to the study endpoints, are reported in Supporting Table S1.

**TABLE 1:** Baseline characteristics of the study cohort.

Variable	Category	Value
Sex (*n* = 45 patients)	Female	23 (51.1%)
	Male	22 (48.9%)

Affected eye (*n* = 48 eyes)	Right	30 (62.5%)
	Left	18 (37.5%)

Etiology	Idiopathic	44 (91.7%)
	Trauma	2 (4.2%)
	After retinal detachment	2 (4.2%)

Gass stage	Stage 2	3 (6.3%)
	Stage 3	22 (45.8%)
	Stage 4	23 (47.9%)

Lens status	Phakic, no significant cataract	28 (58.3%)
	Pseudophakic	9 (18.8%)
	Cataract present	11 (22.9%)

Age, years	Mean ± SD (range)	68.4 ± 8.7 (28–81)

Symptom duration, months	Mean ± SD (range)	6.8 ± 5.3 (2–24)

*Note:* Comorbid systemic conditions are reported in Supporting Table S1.

Abbreviation: SD, standard deviation.

Operative details are given in Table 2. Intraoperative complications occurred in 9 eyes (18.8%): a peripheral retinal break requiring localized laser in 3 eyes (6.3%), multiple peripheral breaks requiring 360‐degree laser in 2 eyes (4.2%), foveal hemorrhage in 2 eyes (4.2%), partial retinal detachment in 1 eye (2.1%), and an iatrogenic break in the macular area in 1 eye (2.1%).

**TABLE 2:** Surgical technique, tamponade, intraoperative complications, and anatomical closure by technique.

Variable	Category	*n* (%)	Closed, *n* (%)	*p* value
ILM management	Complete ILM peeling (no flap)	30 (62.5%)	22 (73.3%)	0.282
	Inverted ILM flap	18 (37.5%)	16 (88.9%)	

Autologous blood	Yes	4 (8.3%)	4 (100.0%)	0.566
	No	44 (91.7%)	34 (77.3%)	

Tamponade	C3F8	41 (85.4%)	32 (78.0%)	1.000
	Air	4 (8.3%)	3 (75.0%)	
	SF6	2 (4.2%)	2 (100.0%)	
	Silicone oil	1 (2.1%)	1 (100.0%)	

Combined phacoemulsification	Yes	11 (22.9%)	9 (81.8%)	1.000
	No	37 (77.1%)	29 (78.4%)	

Intraoperative complication	Yes	9 (18.8%)	7 (77.8%)	1.000
	No	39 (81.3%)	31 (79.5%)	

*Note:* *p* values from the Fisher exact test (Fisher–Freeman–Halton exact test for the tamponade comparison).

Abbreviations: C3F8, perfluoropropane; ILM, internal limiting membrane; SF6, sulfur hexafluoride.

### 3.2. Anatomical and Functional Outcomes

The macular hole was closed after the primary procedure in 34 eyes (70.8%). Of the 14 eyes with a persistent hole, 7 declined further surgery and 7 underwent a second procedure, which achieved closure in 4. Final anatomical success was therefore achieved in 38 eyes (79.2%) and functional success in 36 eyes (75.0%). BCVA at postoperative Months 3 and 6 differed significantly from the preoperative value (both *p* < 0.001). Acuity improved progressively after surgery, and the best values were recorded at Month 6.

Closure did not differ significantly by tamponade agent, use of an inverted ILM flap, use of autologous blood, combined cataract surgery, or the occurrence of an intraoperative complication (Table 2). Sixteen of 18 eyes (88.9%) closed after an inverted ILM flap, against 22 of 30 eyes (73.3%) treated without a flap. The difference favored the flap technique but fell short of significance (*p* = 0.282).

### 3.3. Symptom Duration and Disease Stage

Longer symptom duration was associated with worse BCVA preoperatively and at all postoperative visits (Table 3). The association also strengthened across follow‐up, from *r* = 0.523 preoperatively to *r* = 0.808 at Month 6, so that symptom duration became a progressively better discriminator of visual outcome as recovery proceeded. Symptom duration was also significantly longer in eyes that failed to close (*p* < 0.001). On ROC analysis, symptom duration discriminated anatomical closure with an AUC of 0.963 (95% CI by the DeLong method, 0.918–1.000). At the Youden‐optimal cutoff of 9 months, sensitivity for nonclosure was 100% and specificity 89.5%. Every eye operated within 8 months of symptom onset achieved final closure (34 of 34); of the 14 eyes with a longer symptom duration, only 4 closed. Among the preoperative geometric parameters, only basal hole diameter differed significantly between eyes with and without final closure (959 ± 385 versus 1267 ± 303 μm; *p* = 0.033), and it discriminated closure far less well than duration did (AUC = 0.722). Minimum diameter, hole height, MHI, THI, DHI, and HFF did not differ by closure status (*p* = 0.078–0.517).

**TABLE 3:** Correlation between symptom duration and BCVA (logMAR).

Time point	*r*	*p* value
Preoperative BCVA	0.523	< 0.001
Postoperative Month‐1 BCVA	0.526	< 0.001
Postoperative Month‐3 BCVA	0.697	< 0.001
Postoperative Month‐6 BCVA	0.808	< 0.001

*Note:* Spearman correlation. Higher logMAR values indicate worse visual acuity; a positive *r* therefore denotes an association between longer symptom duration and poorer acuity.

Abbreviations: BCVA, best‐corrected visual acuity; logMAR, logarithm of the minimum angle of resolution.

Stage mattered as well. All 3 Stage 2 holes closed (100%), as did 18 of 22 Stage 3 holes (81.8%) and 17 of 23 Stage 4 holes (73.9%); the small number of Stage 2 eyes precluded formal statistical comparison across stages. More advanced stage was associated with poorer BCVA at baseline (*p* = 0.005). At the postoperative visits, the overall three‐group comparison did not retain significance (*p* = 0.070–0.118), although the ordered trend of poorer BCVA with more advanced stage persisted at every visit (Jonckheere–Terpstra *p* for trend = 0.030–0.045) (Table 4).

**TABLE 4:** BCVA (logMAR) according to the macular hole stage.

Time point	Stage 2 (*n* = 3)	Stage 3 (*n* = 22)	Stage 4 (*n* = 23)	*p* value
Preoperative	0.567 ± 0.115	0.886 ± 0.247	1.122 ± 0.341	0.005
Postoperative Month 1	0.733 ± 0.252	0.873 ± 0.276	1.091 ± 0.349	0.070
Postoperative Month 3	0.667 ± 0.351	0.723 ± 0.289	0.957 ± 0.405	0.118
Postoperative Month 6	0.500 ± 0.200	0.632 ± 0.358	0.835 ± 0.383	0.099

*Note:* Data are mean ± SD. Kruskal–Wallis test; *p* for trend across stages by the Jonckheere–Terpstra test: preoperative, *p* = 0.002; Month 1, *p* = 0.030; Month 3, *p* = 0.045; Month 6, *p* = 0.036. Higher logMAR values indicate worse visual acuity.

### 3.4. Preoperative OCT Biomarkers and Postoperative BCVA

Larger minimum and basal hole diameters went with poorer BCVA, before surgery and at every postoperative visit. Higher MHI and THI values went the other way, with better postoperative acuity. Hole height, DHI, and HFF showed no significant association with postoperative BCVA in the overall cohort (Table 5). The correlation between THI and BCVA weakened progressively over follow‐up (*r* = −0.469 preoperatively to *r* = −0.359 at Month 6), whereas that between basal diameter and BCVA did not (*r* = 0.669 to *r* = 0.521, remaining *p* < 0.001 at every time point).

**TABLE 5:** Correlation of preoperative OCT parameters with BCVA (logMAR).

Parameter	Preoperative	Month 1	Month 3	Month 6
Minimum hole diameter	*r* = 0.538; *p* < 0.001	*r* = 0.459; *p* = 0.001	*r* = 0.423; *p* = 0.003	*r* = 0.389; *p* = 0.006
Basal hole diameter	*r* = 0.669; *p* < 0.001	*r* = 0.499; *p* < 0.001	*r* = 0.506; *p* < 0.001	*r* = 0.521; *p* < 0.001
Hole height	*r* = 0.275; *p* = 0.058	*r* = 0.090; *p* = 0.545	*r* = 0.122; *p* = 0.408	*r* = 0.194; *p* = 0.186
Macular hole index	*r* = −0.659; *p* < 0.001	*r* = −0.546; *p* < 0.001	*r* = −0.517; *p* < 0.001	*r* = −0.527; *p* < 0.001
Tractional hole index	*r* = −0.469; *p* < 0.001	*r* = −0.462; *p* < 0.001	*r* = −0.415; *p* = 0.003	*r* = −0.359; *p* = 0.012
Diameter hole index	*r* = −0.053; *p* = 0.721	*r* = 0.043; *p* = 0.774	*r* = 0.027; *p* = 0.853	*r* = −0.032; *p* = 0.828
Hole form factor	*r* = −0.110; *p* = 0.458	*r* = −0.074; *p* = 0.616	*r* = −0.123; *p* = 0.404	*r* = −0.073; *p* = 0.624

BCVA, best‐corrected visual acuity; OCT, optical coherence tomography. Spearman correlation. Higher logMAR values indicate worse visual acuity; a positive *r* therefore denotes an association with poorer acuity and a negative *r* with better acuity.

### 3.5. Outer Retinal Restoration and Closure Pattern

Eyes with preserved ELM and/or EZ integrity at postoperative Months 3 and 6 had significantly better BCVA than eyes without restoration of these outer retinal layers (all *p* < 0.001) (Table 6). No eye showed restored ELM or EZ continuity at Month 1. At Month 3, ELM continuity was present in 29 eyes and EZ continuity in 18 eyes; at Month 6, the corresponding figures were 29 and 27 eyes.

**TABLE 6:** Postoperative BCVA (logMAR) according to outer retinal integrity.

Time point	Biomarker	Restored	Not restored	*p* value
Month 3	ELM continuity	0.624 ± 0.240	1.147 ± 0.295	< 0.001
Month 3	EZ continuity	0.533 ± 0.178	1.010 ± 0.334	< 0.001
Month 6	ELM continuity	0.472 ± 0.179	1.100 ± 0.258	< 0.001
Month 6	EZ continuity	0.463 ± 0.180	1.052 ± 0.289	< 0.001

*Note:* Data are mean ± SD. Mann–Whitney *U* test. No eye showed restored ELM or EZ continuity at Month 1.

Abbreviations: ELM, external limiting membrane; EZ, ellipsoid zone.

Among the 38 closed holes, 27 eyes (71.1%) showed Type 1 closure and 11 eyes (28.9%) showed Type 2 closure. When preoperative OCT metrics were compared by the closure pattern, HFF was significantly higher in Type 1 closure (0.789 ± 0.163) than in Type 2 closure (0.641 ± 0.190; *d* = 0.86; 95% CI for the mean difference, 0.024 to 0.272; *p* = 0.036), whereas the remaining parameters did not differ significantly (Table 7). The nonsignificant comparisons were not null, however. Their effect sizes were small to moderate (basal diameter *d* = 0.65, minimum diameter *d* = 0.47, MHI *d* = 0.40), and their 95% CIs were wide (basal diameter, −513.0 to 28.6 μm; minimum diameter, −261.8 to 55.0 μm; MHI, −0.143 to 0.502), so that each interval included both no difference and a difference of clinically relevant size. Symptom duration is not an OCT parameter, but it too differed sharply by the closure pattern (3.4 ± 1.0 months in Type 1 versus 8.1 ± 3.8 months in Type 2; *d* = 2.16, *p* < 0.001). Preoperative duration was therefore associated not only with whether a hole closed, but also with how completely it closed.

**TABLE 7:** Preoperative OCT parameters according to the closure pattern, with effect sizes and 95% confidence intervals.

Parameter	Type 1 closure (*n* = 27)	Type 2 closure (*n* = 11)	Mean difference (95% CI)	Cohen’s *d*	*p* value
Minimum hole diameter (μm)	390.3 ± 182.7	493.7 ± 291.3	−103.4 (−261.8 to 55.0)	−0.47	0.394
Basal hole diameter (μm)	888.9 ± 334.0	1131.1 ± 460.1	−242.2 (−513.0 to 28.6)	−0.65	0.088
Hole height (μm)	449.7 ± 80.0	434.7 ± 107.5	14.9 (−49.2 to 79.1)	0.17	0.936
Macular hole index	0.624 ± 0.508	0.445 ± 0.201	0.179 (−0.143 to 0.502)	0.40	0.058
Tractional hole index	1.659 ± 1.644	1.186 ± 0.884	0.473 (−0.595 to 1.542)	0.32	0.234
Diameter hole index	0.460 ± 0.191	0.457 ± 0.200	0.003 (−0.137 to 0.144)	0.02	0.872
Hole form factor	0.789 ± 0.163	0.641 ± 0.190	0.148 (0.024 to 0.272)	0.86	0.036
Operculum, *n* (%)	5 (18.5%)	0 (0%)	OR 5.62 (0.29 to 110.8)^∗^	—	0.295
Epiretinal membrane, *n* (%)	16 (59.3%)	5 (45.5%)	OR 1.70 (0.43 to 6.62)^∗^	—	0.491
Cystoid changes, *n* (%)	22 (81.5%)	7 (63.6%)	OR 2.45 (0.55 to 10.95)^∗^	—	0.401

*Note:* Data are mean ± SD unless otherwise stated; the last three rows are counts (%) with odds ratios. *p* values from the Mann–Whitney *U* test. Cohen’s *d* and the 95% CI for the mean difference were computed from the pooled SD. Qualitative features were compared with the Fisher exact test.

Abbreviations: CI, confidence interval; OCT, optical coherence tomography.

^∗^Odds ratios for these rows were estimated with the Haldane–Anscombe correction and are shown in place of Cohen’s *d*, with Cohen’s *d* not applicable (—).

Three qualitative preoperative features were also recorded: an operculum in 9 eyes (18.8%), an epiretinal membrane in 26 eyes (54.2%), and cystoid changes at the hole edge in 38 eyes (79.2%). None of the three distinguished the closure patterns (Table 7). An operculum was present in 5 of 27 Type 1 eyes (18.5%) and in none of the 11 Type 2 eyes (0%; *p* = 0.295), an epiretinal membrane in 59.3% versus 45.5% (*p* = 0.491), and cystoid changes in 81.5% versus 63.6% (*p* = 0.401). None was associated with Month‐6 BCVA (*p* = 0.989, 0.308, and 0.308, respectively) or with Month‐6 EZ restoration. Operculum was the only feature to approach significance against anatomical outcome, being less frequent in eyes that closed than in eyes that did not (5 of 38, 13.2%, versus 4 of 10, 40.0%; odds ratio: 0.24, 95% CI: 0.05 to 1.07; *p* = 0.075).

### 3.6. Multivariable Analysis

In the multivariable linear regression model for Month‐6 BCVA, two covariates remained independently associated with the outcome: preoperative BCVA (*B* = 0.479; 95% CI, 0.270 to 0.688; *p* < 0.001) and Month‐6 EZ integrity (*B* = −0.356; 95% CI, −0.517 to −0.195; *p* < 0.001). Symptom duration (*B* = 0.014; 95% CI, −0.002 to 0.030; *p* = 0.086) and minimum hole diameter (*p* = 0.276) did not (Table 8). The model accounted for 78.3% of the variance in Month‐6 BCVA (adjusted *R*‐squared = 0.763; overall model *p* < 0.001), and all VIF values were below 2.6, so collinearity was not a problem. In the secondary model containing MHI and THI in place of the hole diameters, neither index was independently associated with Month‐6 BCVA (*p* = 0.970 and *p* = 0.190, respectively), while preoperative BCVA and Month‐6 EZ integrity remained significant (adjusted *R*‐squared = 0.761). Restricting the analysis to one eye per patient (*n* = 45) left the direction and significance of every principal association unchanged (preoperative BCVA and Month‐6 EZ integrity, both *p* < 0.001).

**TABLE 8:** Multivariable linear regression model for postoperative Month‐6 BCVA (logMAR).

Predictor	*B* (95% CI)	Standardized beta	*p* value	VIF
Preoperative BCVA (logMAR)	0.479 (0.270 to 0.688)	0.418	< 0.001	1.62
Symptom duration (months)	0.014 (−0.002 to 0.030)	0.200	0.086	2.58
Minimum hole diameter (per 100 μm)	−0.016 (−0.046 to 0.013)	−0.092	0.276	1.36
Month‐6 EZ integrity (restored vs not)	−0.356 (−0.517 to −0.195)	−0.476	< 0.001	2.26

*Note:* Adjusted *R*‐squared = 0.763; overall model *p* < 0.001. The macular hole index and tractional hole index were tested in a separate secondary model because they are algebraic functions of hole height and hole diameter.

Abbreviations: BCVA, best‐corrected visual acuity; CI, confidence interval; EZ, ellipsoid zone; VIF, variance inflation factor.

## 4. Discussion

This study examined preoperative hole morphology, symptom duration, and postoperative outer retinal restoration together after macular hole surgery. Taken singly, the associations we found between hole size, MHI, THI, ELM/EZ restoration, and visual outcome sit comfortably within a large existing literature, and we do not offer them as new. Three features of the combined pattern are reported less often, and they are what this analysis contributes.

First, the determinants of the two outcome domains were dissociable. Anatomical closure was governed principally by the symptom duration and, to a lesser extent, by the hole stage. Among the preoperative geometric indices, only basal diameter differed by closure status, and it discriminated closure far less well than symptom duration (AUC = 0.722 versus 0.963). For acuity, the picture reversed: minimum diameter, basal diameter, MHI, and THI were all significantly associated with BCVA at every time point. Preoperative geometry, then, chiefly tracked how well an eye would see, while preoperative duration tracked whether the hole would close. The distinction matters at the bedside. An eye with a long‐standing but geometrically favorable hole and an eye with a recent but wide hole carry different risks, and a single composite prognosis conflates them.

Second, the prognostic weight of these markers did not hold steady over time. The correlation between symptom duration and BCVA strengthened from *r* = 0.523 preoperatively to *r* = 0.808 at Month 6, while that of THI weakened from *r* = −0.469 to *r* = −0.359 across the same interval. One reading is that indices reflecting instantaneous tractional geometry describe the preoperative state well and lose relevance once traction is surgically relieved, whereas duration stands in for the cumulative photoreceptor injury that sets the achievable visual ceiling. The attenuation of THI over follow‐up has been reported before [19, 21, 22]. The reciprocal strengthening of the duration–acuity relationship over the same window is, to our knowledge, described less often, and it argues against judging prognostic indices at a single postoperative time point. The multivariable model fits this reading: the contribution of symptom duration was largely absorbed once Month‐6 EZ integrity entered the model, as would be expected if duration acts on final acuity mainly through the completeness of outer retinal restoration.

Third, HFF behaved unlike the other indices. It showed no association with BCVA at any time point (all *p* > 0.40), which is at variance with several earlier reports linking it to postoperative acuity [13, 22], yet it was the only preoperative geometric parameter that separated Type 1 from Type 2 closure (*d* = 0.86; *p* = 0.036), with a concordant borderline association with EZ restoration at Month 3 (*p* = 0.055). The explanation may lie in what HFF measures. Computed from the hole arm lengths relative to the basal diameter, it encodes the slope and apposability of the hole edges rather than the extent of tissue lost. Our data are consistent with HFF indexing the mechanical ease with which those edges can be reapposed, and hence the structural quality of closure, rather than the photoreceptor reserve that determines acuity. This dissociation should be read as hypothesis‐generating and needs confirmation in a larger, prospectively measured series.

That larger holes recover less vision is biologically unsurprising and consistent with prior OCT‐based studies. A wider minimum or basal diameter reflects more advanced tissue loss, broader photoreceptor disruption, and greater retinal displacement, any of which may limit postoperative reconstitution of the foveal architecture [10–13]. In our cohort, both diameters held their association with BCVA from the preoperative visit through Month 6, which supports their use in routine preoperative counseling.

Hole height alone predicted nothing. Several earlier reports point the same way: The vertical configuration on its own does not capture the biomechanical and structural complexity of the lesion [12, 16, 18–21, 28]. Composite indices that combine more than one geometric component are therefore likely to be more informative than any single linear dimension.

The outer retinal bands told a consistent story. Eyes with preserved ELM and EZ continuity at Months 3 and 6 saw substantially better than eyes without restoration. These bands reflect photoreceptor alignment and integrity, so their recovery plausibly supplies the structural substrate for visual rehabilitation. Earlier work points the same way, linking postoperative reconstruction of the photoreceptor‐related layers to functional gain after macular hole closure [16, 17]. Since the relationship is physiologically expected, we treat it here as internal validation of the outer retinal grading rather than as a novel finding and have kept its discussion brief.

The three qualitative features behaved differently from one another in the literature, and our null results should be read against that background. Cystoid change is the best studied of the three. Joo et al. found intraretinal cyst height to differ between Type 1 and Type 2 closure [29], and Ozturk et al. derived cut‐off values for cyst area indices predicting the closure pattern [30]. Both measured cysts quantitatively. Our dataset recorded only the presence or absence, a considerably cruder variable, and the absence of an association here should not be read as contradicting those reports. The epiretinal membrane is confounded by design in our series, since the ILM and any epiretinal tissue were peeled in every eye; the variable therefore describes a feature that was removed at surgery rather than one left to influence healing. Operculum is the least studied of the three against the closure pattern. Larsson et al. examined operculum on OCT in 61 eyes and found no relationship with visual acuity or with the anatomical closure rate [31], but they did not classify the closure pattern, and we are not aware of a series that has. In our cohort, no Type 2 eye had an operculum, and operculum was about three times more frequent among eyes that failed to close. Neither comparison reached significance, and the closure pattern comparison rests on a zero cell in a group of 11, so the observation is hypothesis‐generating only. It is, nonetheless, mechanistically coherent: an operculum marks tissue that has already been avulsed from the fovea, and a hole that has lost its lid may be one in which full‐thickness tissue loss is complete. Whether this holds in a series large enough to test it remains open.

### 4.1. Anatomical Closure Rate in Context

Our final closure rate of 79.2% (38/48) is below the 90%–98% commonly reported in contemporary series, and several features of this cohort account for the gap. The study spans 2013 to 2019, a period over which both surgical experience and vitreoretinal instrumentation at our center changed substantially. Closure was achieved in 7 of 13 eyes (53.8%) operated before January 2017, against 31 of 35 eyes (88.6%) operated thereafter (*p* = 0.016); the later figure sits squarely within the contemporary range. The cohort is also weighted toward advanced disease, with 45 of 48 eyes (93.8%) at Stage 3 or 4 and a mean basal diameter of 1023 ± 387 μm. The mean symptom duration was 6.8 months, and individual eyes presented as late as 24 months after symptom onset. Four eyes (8.3%) had a secondary etiology, traumatic or postdetachment. One further point deserves emphasis: the inverted ILM flap was used in only 18 eyes (37.5%) rather than across the cohort, and the majority underwent conventional ILM peeling. Closure among flap‐treated eyes was 88.9%, consistent with published flap series [7, 32], whereas the pooled figure reflects the mixed technique of a real‐world consecutive series operated over 7 years [33, 34].

### 4.2. Precision of the Closure Pattern Comparisons

Several comparisons of preoperative parameters between closure patterns did not reach significance despite effect sizes that were not negligible. With 27 Type 1 and 11 Type 2 eyes, the estimates were imprecise. The mean difference in basal diameter was −242.2 μm (95% CI, −513.0 to 28.6), in minimum diameter −103.4 μm (95% CI, −261.8 to 55.0), and in MHI 0.179 (95% CI, −0.143 to 0.502). Each interval includes zero, but each also extends to differences that would be clinically meaningful, so the data neither establish nor exclude an association between these parameters and the closure pattern. For the same reason, formal equivalence testing would not be informative here: no clinically negligible margin lies wholly within intervals this wide, so nonequivalence could not be rejected either. The distribution of MHI limits inference further: its range extended to 3.036 against a mean of 0.537 ± 0.403, a pronounced right skew driven by a handful of eyes with very small basal diameters, which blunts the sensitivity of rank‐based comparison. We therefore regard these comparisons as inconclusive and report them with effect sizes and CIs so that they can feed future pooled analyses instead of being read as negative findings.

Longer symptom duration and more advanced stage each went with poorer outcomes, which places a practical premium on timely referral. Operating earlier may limit irreversible photoreceptor damage and improve the odds of both anatomical closure and visual recovery. Historical surgical series reached the same conclusion [35, 36].

### 4.3. Strengths and Limitations

The study has real strengths: a consecutive single‐center cohort with fixed follow‐up intervals, masked OCT measurements, and parallel assessment of anatomical and functional endpoints alongside serial outer retinal grading. The limitations are equally clear. A retrospective design admits selection bias. The cohort was modest in size, and, as set out above, the confidence intervals of several comparisons were too wide to be conclusive. Surgical technique was not uniform. The inverted ILM flap was used in 37.5% of eyes and tamponade agents varied, which reflects real‐world practice across a 7‐year period but limits attribution of outcomes to any single technique; the subgroups defined by technique are too small for reliable comparison. Twelve eyes underwent combined phacoemulsification. The magnitude of BCVA improvement from baseline to Month 6 did not differ between eyes with and without combined cataract surgery (0.25 ± 0.19 versus 0.26 ± 0.31 logMAR; *p* = 0.781), but a contribution of lens status to the measured visual improvement cannot be excluded outright, and progression of lens opacity in the eyes that remained phakic may have worked in the opposite direction, damping the measured gains. Three patients contributed both eyes; a one‐eye‐per‐patient sensitivity analysis left the findings intact, yet residual within‐patient correlation remains possible. Closure pattern was not used as an independent predictor of acuity, since its operational definition and the ELM/EZ variable share an anatomical substrate. All OCT measurements were performed by a single masked observer, and no intra‐ or interobserver repeatability data are available, so observer‐dependent measurement variability cannot be quantified. Idiopathic cases dominated the etiologic distribution, which may limit generalizability to traumatic or secondary macular holes. The multivariable model was necessarily restricted to four covariates and should be read as exploratory. External validation in a larger prospective cohort is required before any of these parameters is used for individual risk stratification.

## 5. Conclusion

Surgery closed most holes in this cohort and improved vision steadily over 6 months. Preoperative OCT biomarkers, particularly minimum hole diameter, basal hole diameter, MHI, and THI, were associated with postoperative BCVA, and postoperative restoration of the ELM and EZ was strongly related to functional recovery. Beyond confirming these established associations, our data point to three things: the determinants of anatomical and functional success are largely dissociable, with symptom duration dominating closure and preoperative geometry dominating acuity; the prognostic weight of individual indices shifts across the postoperative course; and HFF relates to the structural quality of closure rather than to visual acuity. Used together, these parameters can sharpen the prognosis offered to a patient before surgery, although confirmation in larger prospective cohorts is required.

## Funding

The authors received no specific funding for this work.

## Conflicts of Interest

The authors declare no conflicts of interest.

## Supporting Information

Additional supporting information can be found online in the Supporting Information section.

## Supporting information


**Supporting Information** Supporting Table S1: Comorbid systemic conditions in the study cohort.

## Data Availability

The datasets generated and/or analyzed during the current study are available from the corresponding author on reasonable request.
