# Supplementary material for: Optical Coherence Tomography Predictors of Anatomical and Functional Recovery After Macular Hole Surgery
Source: J Ophthalmol. 2026 Oct 8;2026:5093898. doi: 10.1155/joph/5093898 (PMC13646894; doi:10.1155/joph/5093898)
Supplement: Supplementary file 1 [file JOPH-2026-5093898-s001.docx]

**Supplementary Materials**

**Optical Coherence Tomography Predictors of Anatomical and Functional Recovery After Macular Hole Surgery**

**Supplementary Table S1. Comorbid systemic conditions in the study cohort (n = 48 eyes of 45 patients).**

| **Variable** | **Category** | **n (%)** |
| --- | --- | --- |
| Any comorbidity | Present | 39 (81.3%) |
|  | Absent | 9 (18.8%) |
| Specific condition* | Hypertension | 27 (56.3%) |
|  | Goiter | 11 (22.9%) |
|  | Diabetes mellitus | 8 (16.7%) |
|  | Benign prostatic hyperplasia | 6 (12.5%) |
|  | Coronary artery disease | 5 (10.4%) |
|  | Laryngeal cancer | 1 (2.1%) |

** Conditions may coexist in the same patient; percentages therefore sum to more than 100%. No comorbidity was significantly associated with anatomical closure (P = 0.370) or functional success (P = 0.199).*
